# Validation of a Fast, Robust, Inexpensive, Two-Tiered Neonatal Screening Test algorithm on Dried Blood Spots for Spinal Muscular Atrophy

**DOI:** 10.3390/ijns5020021

**Published:** 2019-05-15

**Authors:** Annuska Strunk, Andre Abbes, Antoine R. Stuitje, Chris Hettinga, Eline M. Sepers, Reinier Snetselaar, Jan Schouten, Fay-Lynn Asselman, Inge Cuppen, Henny Lemmink, W. Ludo van der Pol, Henk Engel

**Affiliations:** 1 Department of Clinical Chemistry and Neonatal Screening, Isala Hospital, Dokter van Heesweg 2, 8025 AB Zwolle, The Netherlands; 2 MRC-Holland, Willem Schoutenstraat 1, 1057 DL Amsterdam, The Netherlands; 3 Department of Neurology, Brain Center Rudolf Magnus, University Medical Center Utrecht, Universiteitsweg 100, 3584 CG Utrecht, The Netherlands; 4 Department of Genetics, University Medical Center Groningen, Antonius Deusinglaan 1, 9713 AV Groningen, The Netherlands

**Keywords:** newborn screening, SMA, melting curve, two-tiered testing, copy numbers *SMN1* and *SMN2* gene

## Abstract

Spinal muscular atrophy (SMA) is one of the leading genetic causes of infant mortality with an incidence of 1:10,000. The recently-introduced antisense oligonucleotide treatment improves the outcome of this disease, in particular when applied at an early stage of progression. The genetic cause of SMA is, in >95% of cases, a homozygous deletion of the survival motor neuron 1 (*SMN1*) gene, which makes the low-cost detection of SMA cases as part of newborn screening programs feasible. We developed and validated a new SALSA MC002 melting curve assay that detects the absence of the *SMN1* exon 7 DNA sequence without detecting asymptomatic carriers and reliably discriminates *SMN1* from its genetic homolog *SMN2* using crude extracts from newborn screening cards. Melting curve analysis shows peaks specific for both the *SMN1* gene and the disease modifying *SMN2* homolog. The detection of the *SMN2* homolog, of which the only clinically relevant difference from the *SMN1* gene is a single nucleotide in exon 7, was only used to confirm a correct reaction in samples that lacked the *SMN1* gene, and not for *SMN2* quantification. We retrieved 47 DBS samples from children with genetically-confirmed SMA, after informed consent from parents, and 375 controls from the national archive of the Dutch National Institute for Public Health and the Environment (RIVM). The assay correctly identified all anonymized and randomized SMA and control samples (i.e., sensitivity and specificity of 100%), without the detection of carriers, on the three most commonly-used PCR platforms with melting curve analysis. This test’s concordance with the second-tier ‘golden standard’ P021 SMA MLPA test was 100%. Using the new P021–B1 version, crude extracts from DBS cards could also be used to determine the *SMN2* copy number of SMA patients with a high level of accuracy. The MC002 test showed the feasibility and accuracy of SMA screening in a neonatal screening program.

## 1. Introduction

Spinal muscular atrophy (SMA) is a leading genetic cause of infant mortality [1]. SMA is an autosomal recessive disease, characterized by muscle weakness and atrophy, caused by the degeneration of the neuronal cells in the anterior horn of the spinal cord [2]. In the vast majority of patients, SMA is caused by a homozygous deletion of at least exon 7 of the survival motor neuron 1 (*SMN1*) gene [3,4]. The human genome also contains a highly identical *SMN1* gene homologue, called *SMN2* [3,5]. The exonic sequence of the *SMN1* and *SMN2* genes differs only by two nucleotides, of which the C-to-T substitution in exon 7 is critical, as it leads to the omission of exon 7 in the majority of transcripts due to splicing events [6,7]. Exon 7 is spliced out of up to 90% of *SMN2* mRNAs, resulting in a non-functional protein [8,9]. The remaining 10% of *SMN2* mRNAs however, are spliced correctly, resulting in a full-length protein that is identical to *SMN1*. Therefore, in SMA patients lacking a functional *SMN1* gene, a small, yet insufficient, amount of functional SMN protein is produced. The *SMN2* copy number varies between patients, and a higher gene copy number is correlated to a milder phenotype [10].

Recently, a therapeutic agent, nusinersen (Spinraza™), has become available. Nusinersen is an antisense oligonucleotide that modulates alternate splicing of *SMN2*, leading to the inclusion of exon 7, thereby increasing the amount of functional SMN protein [11]. Clinical trials have shown that the administration of nusinersen increases the number of motor milestones achieved [12,13], although the cost-effectiveness and long-term benefits are still under debate. For optimal performance, nusinersen should be administered presymptomatically, preferably shortly after birth [14]. It therefore becomes increasingly important to screen neonates as soon as possible for SMA, preferably as part of a nationwide neonatal screening program. This screening method should furthermore reliably detect the *SMN2* copy number, as this determines the neonates’ eligibility for nusinersen administration [14]. A rapid, reliable, and cost-effective screening method is therefore needed to accommodate the high throughput of samples.

We developed a simple melting curve-based assay for SMA newborn screening that only requires a qPCR instrument and standard laboratory equipment. A single probe is used to generate three potential melt peaks: A peak each for *SMN1* and *SMN2*, and a DNA quantity peak (Q-fragment). An absence of the *SMN1* peak identifies SMA patients, and the *SMN2* peak provides an internal control for a successful reaction, as complete absence of *SMN1* and *SMN2* is lethal and every individual has at least one *SMN1* or one *SMN2* gene copy. The Q-fragment provides a measure for the amount of input-DNA. For independent confirmation of the absence of *SMN1*, and for *SMN2* copy number determination, the MLPA technique is used [15,16,17]. In a retrospective study, we tested the accuracy and reliability on DBS (dry blood spot) material of this screening algorithm and show here its feasibility for use in a neonatal screening program.

## 2. Materials and Methods

### 2.1. Samples

For this study, a total of 422 newborn DBS samples were acquired from the national archive of the Dutch National Institute for Public Health and the Environment (RIVM). All samples were originally obtained for the regular Dutch newborn screening program. The SMA samples and the negative controls were both collected from the years 2012–2018 (see Table 1). Parents of the SMA patients born in this time period were asked for written consent for the use of the DBS cards. For all samples used in the present study, written consent was provided. This study was conducted in accordance with the Declaration of Helsinki, and the protocol, with project identification code 17-622/C, was approved by the Medical Ethics Committee of the University Medical Center Utrecht on 6 September 2017. The DBS samples, 47 SMA patients and 375 random negative controls, were anonymized and randomized at the RIVM, and sent to Isala Hospital Zwolle. From each DBS sample, one 1.5 mm and one 3.2 mm punch were collected. The 1.5 mm punches were used for melting curve (MC) analysis, and the 3.2 mm punches were used for MLPA analysis. A blank punch was taken between each sample to reduce the risk of contamination.

For test development and the analytical performance testing of the first-tier melt curve assay, DBS samples were used that were spotted with peripheral blood from a healthy adult control.

### 2.2. Extraction

Extraction was based on a previously-described DNA extraction method for DBS screening cards [18]. Each 1.5 mm punch was immersed in 30 µL of 10 mM NaOH in a 96-well PCR plate and heated for 15 min at 99 °C in a regular thermocycler with a heated lid. The crude extracts obtained could be used for the first-tier MC002 test without further purification. For the second-tier MLPA assay, extracts from the 3.2 mm punches were used. Each punch was first incubated for 15 min at room temperature (RT) in 100 µL of 10 mM NaOH in a PCR plate with intermittent gentle shaking. After discarding the liquid, this wash was repeated. Finally, the punches were heated for 15 min at 99 °C in 50 µL 10 mM NaOH in a regular thermocycler with a heated lid, and the crude extracts obtained from this process were used for MLPA analysis without further purification.

### 2.3. Screening

**Figure 1:**
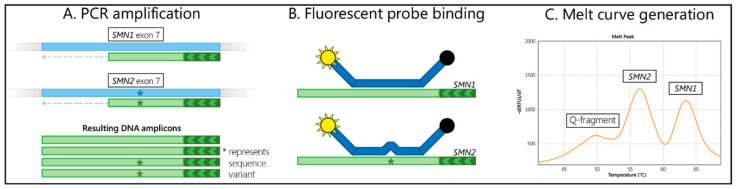
Summary of assay steps. (**A**) The exon 7 regions of *SMN1* and *SMN2* are amplified with a single set of primers, with one primer in excess. (**B**) A fluorescently-labelled probe binds to the amplicons. (**C**) The resulting melt curve indicates the *SMN1* and *SMN2* sequence presence and DNA quantity.

The DBS samples were screened using a two-tiered approach. In the first-tier, the absence of *SMN1* exon 7 was detected with a melt curve assay, branded SALSA^®^ MC002 SMA Newborn Screen (MC002 SMA) (MRC–Holland, Amsterdam, The Netherlands). The MC002 SMA assay contained a primer set that asymmetrically amplified a part of exon 7 that included the c.840C>A polymorphism, which distinguished *SMN1* exon 7 from *SMN2* exon 7. PCR amplification of exon 7 of the *SMN1* and *SMN2* genes was followed by a Cy5-labelled fluorescent probe, binding to the amplicons and generating a melt curve (Figure 1). The fluorescence was only measured during the melt curve generation. The assay also contained an oligonucleotide that comprised the same SMN-specific region, but which contained multiple mismatches with the probe, and therefore generated a third melting curve pattern. This oligonucleotide was titrated to produce a peak only when a very small amount of input-DNA was used. Absence of the *SMN1*-specific melt peak at 63 °C was indicative of the absence of the *SMN1* exon 7 DNA sequence. The presence of an *SMN1* (63 °C) and/or *SMN2* (56 °C)-specific melt peak, and an absent or low signal for the quantity (Q)-fragment-specific melt peak (49 °C) indicated a successful assay performance and the use of sufficient sample DNA. In each 96-well microtiter plate run, two reactions on blank DBS cards were included (containing no DNA). Furthermore, two reactions were included on each of the two synthetic DNA samples (SD074 and SD075) that were included in each MC002 SMA kit.

The SD074 threshold DNA sample had a ratio *SMN1*:*SMN2* = 1:5 and generated a high *SMN2*-specific melt peak at ~56 °C and a low, but clearly visible, *SMN1*-specific melt peak at ~63 °C. The SD075 positive DNA sample generated a high *SMN2*-specific melt peak and had a complete absence of the *SMN1*-specific melt peak.

The melt curve assays were performed on the LightCycler 480 (Roche Molecular Systems, Pleasanton, CA, USA), CFX96 Touch (Bio–Rad, Hercules, CA, USA), and QuantStudio 5 (Thermo Fisher Scientific, Waltham, MA, USA) instruments.

In the second-tier MLPA assay, the absence or presence of the *SMN1* exon 7 was confirmed in all positive samples and negative samples, using the MLPA kit P021–B1 SMA (MRC–Holland), according to the manufacturer’s instructions and using 5 µL crude extract from a 3.2 mm punch.

### 2.4. Input Range

To test the input range of MC002, a total of 3 µL of adult blood was pipetted on 1.5 mm DBS card punches. The 3 µL blood was either not diluted (saturated), or 4, 6 and 8 times diluted in advance. For a measurement of oversaturation of a DBS card, 6 µL of blood was pipetted on the DBS card punches. In addition to the oversaturated samples, four decreasing amounts of input blood were also tested: saturated, 4 times diluted, 6 times diluted, and 8 times diluted. Four replicates were tested for each amount of input blood sample.

### 2.5. Data Analysis

For each melt curve, the first derivative was obtained and inspected. The results of MC002 SMA reactions were examined through visual inspection by two individuals. The reactions with a clear *SMN2* peak at 56 °C, but lacking an *SMN1*-specific peak at 63 °C or with an *SMN1*-specific peak that was lower than the peak in the SD074 threshold DNA reactions, were selected for subjection to the second-tier MLPA test. The reactions were repeated when the Q-fragment melt peak at 49 °C was the highest peak present. Dedicated data analysis software was not yet available at the time of these validation experiments. For the MLPA assays, Coffalyser.Net software (MRC–Holland, Amsterdam, The Netherlands) was used for data analysis.

## 3. Results

### 3.1. Melt Curve Assay Screening Data

**Figure 2:**
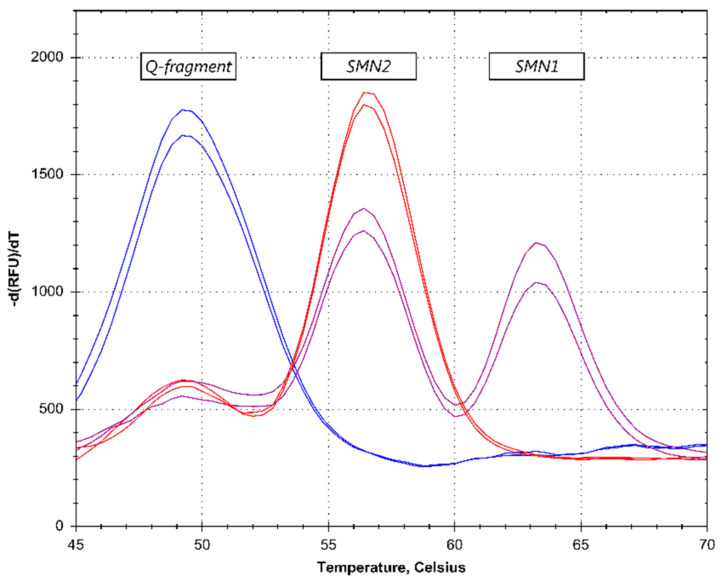
Patient identification—melting curve (MC) results. The purple lines represent normal samples, as determined by the presence of an *SMN1* peak. The red lines show an absence of an *SMN1* peak, identifying a spinal muscular atrophy (SMA) patient. The blue lines represent no DNA control reactions, which results in a high quantity (Q)-fragment. The profiles were acquired on the CFX96 Touch PCR-platform.

The melt curve profiles of a total of 422 anonymized and randomized samples were analyzed. Samples were considered SMA-positive when the *SMN1* peak pattern was absent in the presence of an *SMN2* pattern (Figure 2).

Of the 422 samples, 47 samples were found to lack copies of the *SMN1* exon 7, which indicates a potential SMA patient. All 422 samples were further analyzed using the MLPA kit P021–B1 SMA. The MLPA results entirely matched the melt curve results. All results also matched the original diagnosis. Table 2 provides an overview of these results.

**Figure 3:**
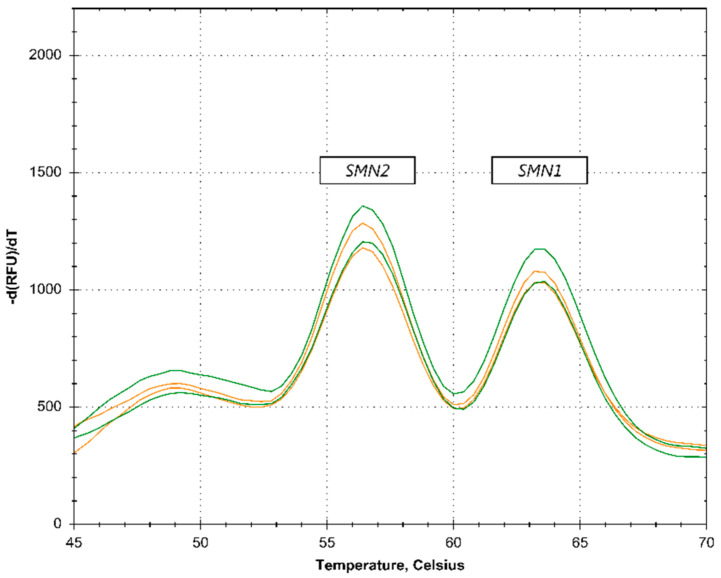
No carrier identification. The orange lines represent samples containing 1 copy of *SMN2* and 1 copy of *SMN1* (SMA carrier). The green lines represent samples containing 2 copies of *SMN2* and 2 copies of *SMN1*. The profiles were acquired on the BioRad CFX96 PCR-platform.

On the LightCycler 480 instrument, out of the 422 samples tested, 7 samples needed to be retested due to unreliable initial results (such as a high Q-fragment, see Figure 2). Upon retesting, the results for these samples could be interpreted. On the CFX96 Touch, two samples needed to be retested and on the QuantStudio5, no samples needed retesting. SMA carriers were not detected in the first-tier analysis (Figure 3).

### 3.2. Input Range

**Figure 4:**
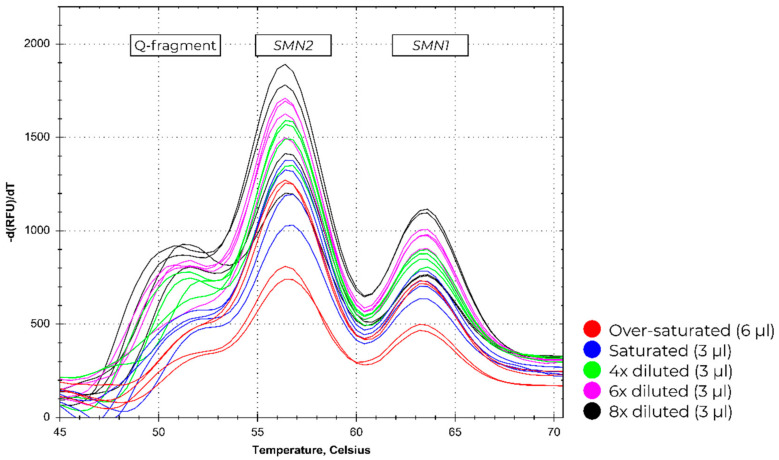
The melting curve profiles for different amounts of DBS cards derived from input blood. Profiles were acquired on the BioRad CFX96 PCR-platform.

To test the input range of MC002, increasingly-lower amounts of input DNA were analyzed using MC002 for spotting diluted blood samples on DBS cards. The increasingly lower amount of input DNA is reflected in the increasing fluorescent signal of the Q-fragment peak (Figure 4). Next to the diluted samples, oversaturated DBS cards were analyzed. Due to the quenching effect that blood has on the fluorescent signal of the Cy5 fluorochrome, lower signals were present in the oversaturated sample compared to the diluted samples, despite more DNA being present.

### 3.3. SMN2 Copy Number Determination

As well as confirming of the absence of the *SMN1* exon 7, the second-tier MLPA method was also used for *SMN2* copy number determination on the DBS samples. The MLPA kit P021–B1 SMA was used for this, as it is best suited with its higher accuracy for *SMN2* copy number determination compared to the widely-used P060–B2 MLPA assay, due to the inclusion of seven additional probes for the *SMN1*/*2* exons 7 and 8. All negative controls were also tested by MLPA. Table 3 provides an overview of the *SMN2* copy number as determined by MLPA. After completion of all validation experiments, *SMN2* copy numbers of SMA patients were compared with the *SMN2* copy numbers previously determined in peripheral blood for diagnostic purposes by the University Medical Centre Utrecht (UMCU). For 3 out of 43 SMA patients, a different *SMN2* copy number was found between the UMCU results and the present validation study. For four out of 47 SMA patients, no *SMN2* data were available at the UMCU.

## 4. Discussion

This study shows that by using a two-tiered system, SMA patients can be detected with a very high reliability and at low costs in a neonatal screening program. The second-tier P021–B1 MLPA assay can also provide early *SMN2* copy number determination from DBS, to determine eligibility for treatment.

The first-tier melt curve assay, MC002 SMA, showed an analytical sensitivity and specificity of 100% for the detection of homozygous *SMN1* exon 7 deletions. This assay can provide results within a few hours after receiving a DBS card, requiring a minimal amount of input-DNA. In one run, 90 samples can be tested with up to 2–3 runs per day on a single PCR platform, allowing for the high throughput of samples that is required for screening purposes. MC002 did not require DNA purification and is in compliance with Dutch regulations concerning neonatal screening, as it entails a qualitative approach that does not detect carriers. By designing the *SMN1* peak to have the highest melt temperature, the risk of false negatives as a result of a genetic variation-induced shift of peaks is mitigated. Genetic variation in the sequence detected by the probe will always result in a lower melting temperature. Genetic variation in the PCR primer sequence may result in a false positive, but never in a false negative screening result. The number of samples that qualify for the second-tier MLPA test is very low. Out of 375 control samples used in this survey, none qualified for the second-tier test.

This retrospective validation study correctly identified all anonymized SMA and control samples. It should be kept in mind, however, that simple first-tier screening assays like MC002 are not able to detect rare SMA-causing genetic aberrations other than the homozygous deletion of exon 7. Early detection by DNA analysis of these patients is extremely complicated, due to the *SMN2* pseudogene, and not feasible as part of a newborn screening program. In practice, this means that 2–5% of SMA patients will not be detected by the two-tiered melting curve and MLPA systems described here.

The second-tier MLPA assay, P021–B1, was also shown to have a diagnostic sensitivity and specificity of 100% for the detection of homozygous exon 7 deletions using crude lysates from DBS cards. Second-tier MLPA testing thereby highly reduces the risk of detecting false positives. Based on results obtained during this clinical performance study, it was shown that the P021–B1 assay can also be reliably used for *SMN2* copy number quantification on DBS material. This *SMN2* copy number detection is important for determining the eligibility of SMA patients for nusinersen treatment [14]. The MLPA retests were generally performed when the *SMN2* copy number results indicating more than two copies were ambiguous due to the sample quality. No MLPA reactions had to be repeated to confirm the absence of *SMN1* exon 7 copies.

As per Dutch regulation [19], the first-tier melting curve assay was designed to minimize detection of carriers, as carrier detection can potentially harm families [20]. This was achieved by using a single probe for the detection of both *SMN1* and *SMN2*, which only indicated the ratio between the copy number of these two genes. This excluded the detection of carriers with an *SMN1*:*SMN2* ratio comparable to non-carriers (i.e., 1:1 vs. 2:2, or 1:2 vs. 2:4, see Figure 2). Carriers with high numbers of *SMN2* copies could potentially stand out upon visual inspection. However, carrier status cannot be definitively determined without follow-up testing, and only the very rare samples with an *SMN2*:*SMN1* ratio equal to or higher than five qualified for the second-tier MLPA test.

Several methods have been tested for their suitability as newborn screening assays. Previously-described approaches include real-time quantitative PCR [21,22], droplet digital PCR [23], competitive oligonucleotide priming PCR [24,25], and next generation sequencing [26]. A major difference is that, in contrast to other assays, Melt Assay MC002 does not detect carriers. Furthermore, it differs from other methods in its cost-effectiveness (€ 2.50–€ 4.00 per sample), requirement of a very simple DNA extraction without washing steps, easy-to-use protocol, and short turn-around time. Furthermore, only a small amount of material, a 1.5 mm punch, is sufficient for clear results. Alternatively, 2–5% of the DNA sample that is extracted for SCID analysis can be used for this melt assay.

Initially, for 3 out of 43 SMA positive samples, a different *SMN2* copy number was found compared to the copy number found in diagnoses of peripheral blood as performed at the UMCU. Upon reanalysis by the UMCU, the same *SMN2* copy number was found for one SMA patient as detected during the present study. A reanalysis of the two remaining samples is pending at the UMCU.

## 5. Conclusions

The first-tier melt curve MC002 assay in combination with the second-tier P021–B1 MLPA assay showed feasibility and accuracy for SMA screening in a neonatal screening program to detect SMA patients, and can be used for early determination of an *SMN2* copy number. The melt curve MC002 and the P021–B1 MLPA assays are CE–IVD-certified as of October 2018, and are Research Use Only assays outside of the European Union.

## Figures and Tables

**Table 1:** Characteristics of DBS cards.

Year of Birth	SMA Patients	Negative Controls
2012	12	75
2013	13	50
2014	6	50
2015	7	50
2016	4	50
2017	3	50
2018	2	50
Total:	47	375

**Table 2:** The number of SMA positive and negative control samples tested using MC PCR-platform and the MLPA kit P021–B1.

	Sample Count	MC—LightCycler 480	MC—CFX96 Touch	MC—QuantStudio 5	MLPA—P021
SMA positive samples	47	47	47	47	47
Negative controls	375	375	375	375	375
Retested samples		5 (1.2%)	2 (0.5%)	1 (0.2%)	17 (4.0%)

MC: Melting curve, MLPA: Multiplex ligase dependent probe amplification.

**Table 3:** *SMN* copy number for all tested DBS samples.

		*SMN2* exon7					
		0	1	2	3	4	Total
***SMN1*** **exon7**	0	0	1	13	27	6	47
	1	0	3	2	4	0	9
	2	24	135	165	7	1	332
	3	3	14	12	1	0	30
	4	2	1	1	0	0	4
	Total	29	154	193	39	7	422
